# Dramatic increase in volume versus length of invasive ductal carcinoma mimicking intramammary lymph node in a small nodular lesion

**DOI:** 10.1186/s42269-022-00823-y

**Published:** 2022-05-12

**Authors:** Seda Aladag Kurt, Varol Celik

**Affiliations:** 1 Department of Radiology, Cerrahpasa Faculty of Medicine, Istanbul University-Cerrahpasa, Kocamustafapasa Street, Fatih, Istanbul, 34098 Turkey; 2 Department of General Surgery, Cerrahpasa Faculty of Medicine, Istanbul University-Cerrahpasa, Istanbul, Turkey

**Keywords:** Breast cancer, Screening, Mammography, Intramammary lymph node, 3D

## Abstract

**Background:**

The tumor growth pattern in breast cancer appears to be variable and unpredictable. A minor increase in size in a pre-existing lesion, especially under 1 cm, corresponds to a more pronounced increase in volume.

**Case presentation:**

We report a 63-year-old woman with a nodular density mimicking intramammary lymph node, diagnosed as invasive carcinoma of luminal B subtype. The lesion increased size and density over time in following mammograms until having indistinct margins. While the tumor volume was 12.7 mm^3^ at the first observation on mammography, it has increased approximately 6.7 times to reach 85.7 mm^3^ in four years. Finally, the patient diagnosed with early-stage breast cancer (T1N0M0) was treated with breast-conserving surgery.

**Conclusions:**

Minor changes in size, density, and margin status of a lesion on serial mammograms are warning for breast cancer. Withal, a slight increase in lesion size in two dimensions can result in significant differences in volume. Therefore, comparative evaluation with previous mammograms and observing any difference in morphological features by screening are crucial for early diagnosis and optimal management of the disease.

## Background

Although breast cancer can be detected early by screening, it is the most common cause of death, especially in the pre-perimenopausal periods (Bray et al. 2021; Dogan et al. 2020). The most effective step to fight breast cancer is screening by mammography. Factors such as the quality of the mammography, the frequency of screening, and the radiologist's experience affect the success of the screening programme. Comparative evaluation with serial mammograms is particularly effective in detecting newly developed pathologies or changes in pre-existing lesions. As the tumor grows in all directions in a three-dimensional (3D) space, changes in volume will be more critical than the increase in size (Mao et al. 2019).

We reported the progressive volumetric enlargement of an invasive breast carcinoma over time that was false negatively evaluated as an intramammary lymph node (IMLN) by regular screening.

## Case presentation

A 63-year-old postmenopausal woman presented to our clinic for annual breast screening. The patient had no family history of breast cancer, or interventional procedure or an operation related to the breast. She had a history of two live births, smoked 20 packs/year, and had no oral contraceptives or hormone replacement therapy. The patient was using levothyroxine only because of hypothyroidism. The patient had no complaints, and the physical examination findings were normal. All mammographic examinations, since 2011, were performed by a digital full-field mammography system (Selenia Dimensions®, Hologic Inc).

**Fig. 1:**
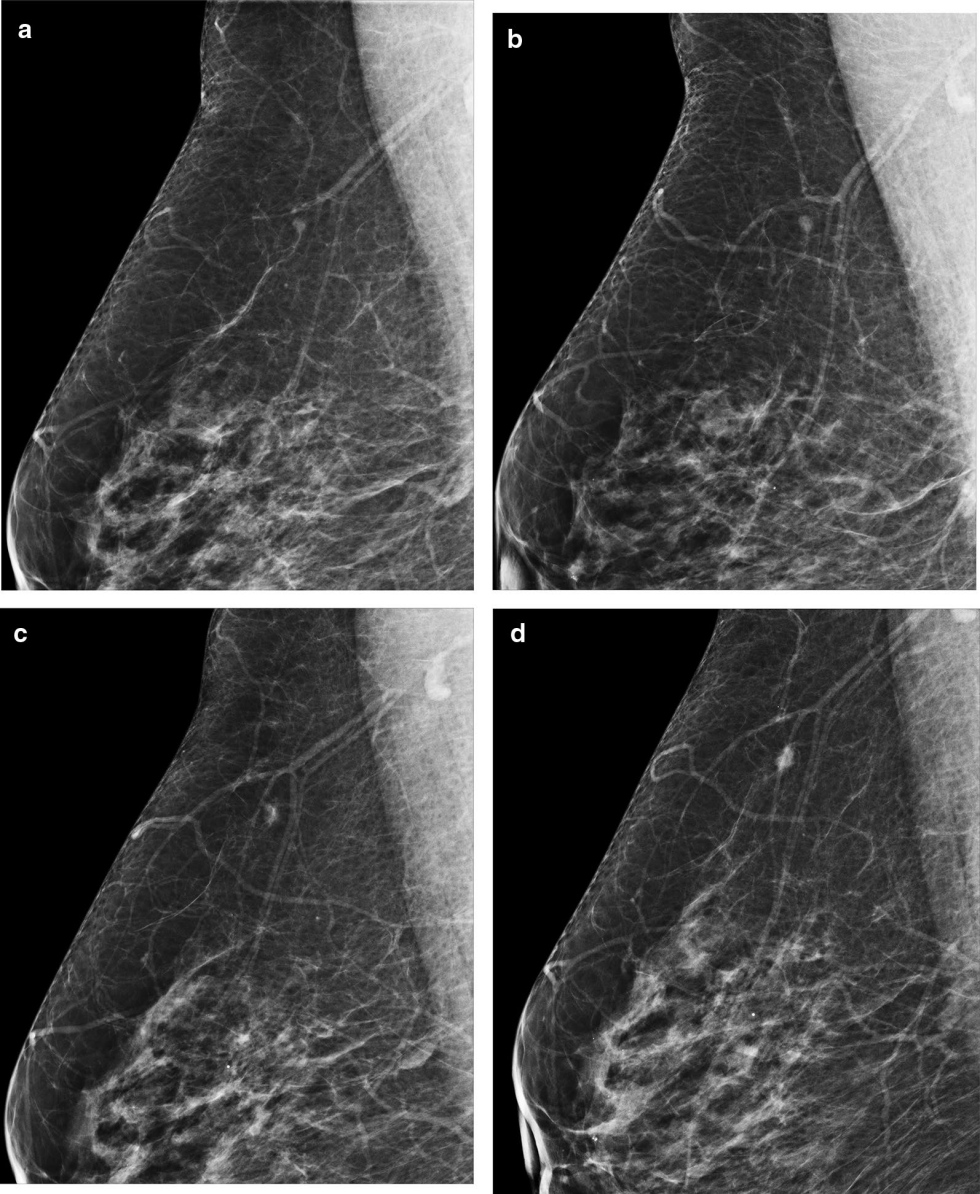
A millimetric density was observed in the upper quadrant of the right breast in annual serial mammograms. **a** A small nodular density mimics an IMLN. **b** Minor difference in size and density. **c** Increase in density and partially obscured margins. **d** Significant increase in size and density, indistinct margins, particularly at the anterior part of the lesion

The breast structure of the patient was in type B pattern. A 3 mm in size nodular opacity with smooth borders was observed in the upper part of the right mediolateral oblique (MLO) radiograph for the first time in 2016 (Fig. 1a). For the following three years, this density was accepted as an intramammary lymph node (IMLN) due to its localization by different radiology residents because of the lack of an expert breast radiologist (Fig. 1b, c). When examined retrospectively, it is noteworthy that this density grows each year in the two-dimensional plane, albeit quite slowly. However, when 2018 was accepted as the breaking point, it was observed that the density of the lesion slightly increased, and the edges of the lesion were no longer smooth but partially obscured (Fig. 1c). When the patient, who did not participate in the screening program because of the COVID-19 pandemic, finally applied in late 2020, it was noted that the lesion became completely hyperdense in addition to enlargement (Fig. 1d). When looked carefully, the lesion borders were indistinct in all sections.

**Fig. 2:**
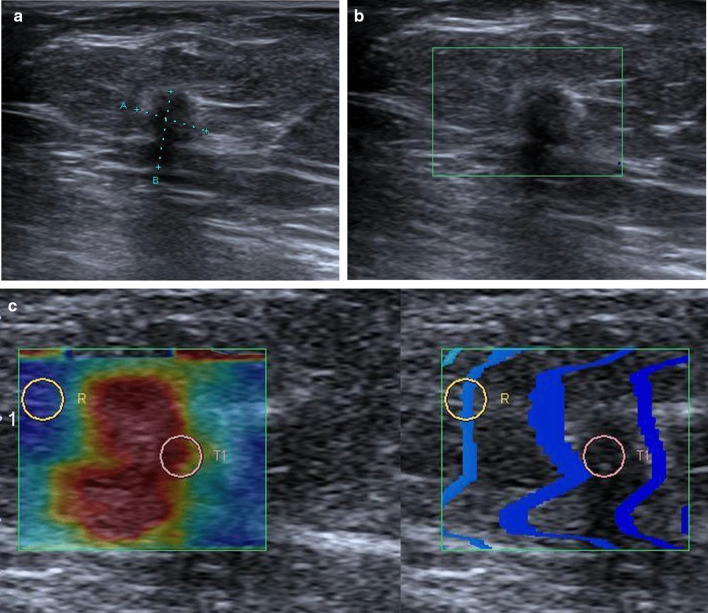
Sonographic findings. **a** The vertically oriented hypoechoic lesion, irregular in shape with indistinct margins. **b** No vascular coding in color Doppler image. **c** High elasticity features with shear wave elastography

A hypoechoic mass lesion was observed in the right breast at the 10 o'clock position on ultrasound (US) examination by a linear transducer (Aplio 500, Toshiba Medical Systems Corporation, Canon Inc, Japan) (Fig. 2a). The borders of the vertically located lesion were irregular and indistinct. The echogenicity of the adjacent parenchyma was slightly increased secondary to desmoplastic reaction. No significant vascularization was observed in the lesion on color Doppler images (Fig. 2b). High elasticity values were obtained simultaneously (Fig. 2c). The lesion evaluated in BI-RADS 5 category was sampled by US-guided core biopsy. The histopathological examination revealed as an invasive ductal carcinoma with nuclear grade 2, positive for estrogen receptor (ER) (100%), positive for progesterone receptor (PR) (10%) and negative for human epidermal growth factor receptor 2 (HER2). The Ki-67 labeling index was 16%.

**Fig. 3:**
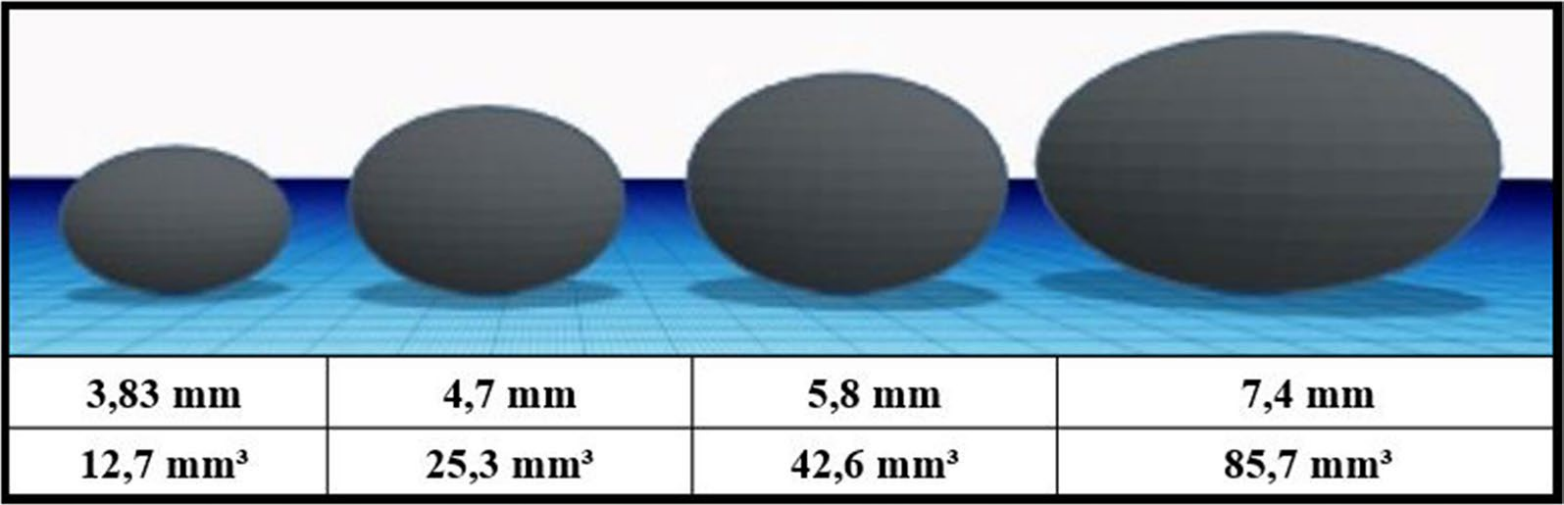
Illustration of volumetric increase relative to serial mammograms. First line: The largest diameter of the density on mammography (mm) Second line: The volumetric measurement of the density (mm^3^)

The experienced breast radiologist measured in consensus the largest tumor diameter on each mammogram using a calibrated built-in software tool (Syngo Mammoreport; Siemens, Erlangen, Germany). Caution was exercised to measure reproducibly, consistently and always in the same projection between the serial mammograms. The choice of the projection was based on where the tumor was most clearly discerned. While the lesion continued to grow quite insidiously over the years in the two-dimensional plane, we calculated how much the lesion enlarged in three dimensions retrospectively (Fig. 3).

The patient was operated on the breast-conserving surgery procedure. No lymphovascular or perineural invasion was detected. Sentinel lymph node biopsy was negative. No peri-postoperative complications developed. The treatment process was completed with chemo- and radiotherapy.

## Discussion

The main purpose of screening is to detect breast cancer at the early stage (Michaelson et al. 2003). Early detection of cancer at a small size directly prolongs life expectancy (Michaelson et al. 2003; Tabar et al. 2000). The meta-analyses based on the high-priority clinical studies have shown that the mortality rate is significantly reduced with regular screening (Smith et al. 2003, 2004). Also, it has been reported in various studies that the prognosis is better in breast cancer diagnosed by screening (Lehtimaki et al. 2011; Maiz et al. 2020; Coldman et al. 2014).

The lesions, mainly located in the upper outer quadrant, can be easily missed by confusing them with IMLN (Bitencourt et al. 2019). In the case we present, the lesion, which has existed for years, was evaluated as an IMLN at first glance by observers who did not have sufficient breast imaging experience. Small size increases in an existing lesion should not be underestimated. A minor difference in size in two-dimensional mammograms corresponds to more significant volume increases. A few studies stated that a tumor grows in a hemi-ellipsoid shape (Mao et al. 2019; Morrison et al. 1983; Faustino-Rocha et al. 2013). In the earlier phase of the cancer tumor grows slowly. However, after reaching a certain size, the growth shows an exponential increase. Although this growth pattern differs in different tumor subtypes, it can be represented by some complex formulas (Mao et al. 2019). It should be noted that tumors in non-aggressive subtypes, such as luminal ones, may grow slowly. We want to emphasize that the increase in size and density is an alerting finding for cancer in the comparative evaluation. And also, marginal features should be magnified and carefully evaluated, especially in sub-centimetric lesions. As Woods et al. pointed out, high-density masses are mostly associated with cancer, as well as findings indicating malignancy characteristically such as round shape, indistinct margins, and increased size (Woods et al. 2011). In addition, comparative evaluation with older mammograms in detecting newly developed pathologies or changes in pre-existing lesions is an indispensable part of screening. Two years and earlier comparisons may be more valuable if available. Although these findings are familiar to the literature, we believe that the highlighted clues are quite instructive and remarkable for all breast professionals from different disciplines. As in this case, a slight increase in lesion size may go unnoticed by the radiologist. However, more significant growth will occur in the 3D universe. Therefore, artificial intelligence algorithms can be used in screening and diagnosis of breast cancer for large case series, and some complex formulas for staging the disease.

## Conclusions

Comparative evaluation is critical in breast imaging, no matter what purpose it is taken for screening or diagnostic purposes. It is valuable to compare with the oldest available images when comparing mammograms. Even a minor increase in size and density in an existing lesion should warn the radiologist. A slight increase in lesion size results in significant differences in the volume. In breast cancer, volumetric measurements may replace traditional methods based on the largest diameter of the breast lesion alone.

## Data Availability

All data used for this report are included in the text.

## Abbreviations

BI-RADS: Breast imaging reporting and data systems
COVID-19: Coronavirus disease of 2019
ER: Estrogen receptor
HER2: Human epidermal growth factor receptor 2
IMLN: Intramammary lymph node
MLO: Mediolateral oblique
PR: Progesterone receptor
TNM: Tumor node metastasis
US: Ultrasound
3D: Three-dimensional
